# Physiologic Events of Embryo Implantation and Decidualization in Human and Non-Human Primates

**DOI:** 10.3390/ijms21061973

**Published:** 2020-03-13

**Authors:** Maria Ariadna Ochoa-Bernal, Asgerally T. Fazleabas

**Affiliations:** 1 Department of Obstetrics, Gynecology & Reproductive Biology, Michigan State University, Grand Rapids, MI 49503, USA; ochoaber@msu.edu; 2 Department of Animal Science, Michigan State University, East Lansing, MI 48824, USA

**Keywords:** cytokines, decidualization, embryo, endometrium, gynecological pathologies, implantation, miRNA, Notch, primates

## Abstract

Reproduction is a fundamental process for the preservation of the human species. This process requires a sequence of orchestrated events that are necessary for a successful pregnancy. Two of the most critical steps in the establishment of human pregnancy are implantation and decidualization, which are required for maternal interactions with the developing embryo. This review primarily highlights the physiological aspects of these two events and the adverse pregnancy outcomes from defective implantation and decidualization. The focus of this review is to provide a general concept of the mechanisms involved during the window of implantation, description of components involved in the process and possible pathologies that could disrupt the embryo implantation and decidualization and specifically as it applies to women and non-human primates.

## 1. Introduction

Successful pregnancy in humans and non-human primates relies on a series of unique events including embryo implantation, decidualization, placentation and parturition. Each of these events are crucial to advance to the next step in pregnancy [1]. Implantation requires an intimate dialogue between the embryo and a receptive endometrium orchestrated by molecular and physiological events. Human implantation is a process that requires essential events such as apposition, adhesion/attachment, invasion and immune regulation [2,3]. These sequential steps lead to a successful pregnancy. Understanding the physiologic mechanisms of the early stages of pregnancy that coordinate pathways for successful embryo implantation and decidualization will significantly improve not only pregnancies conceived naturally but also pregnancies derived from assisted reproductive technologies [4].

## 2. Preimplantation

### 2.1. Fertilization

Fertilization involves fusion of the female (oocyte) and male gametes (sperm). The sperm fertilizes the oocyte creating a single diploid cell, the zygote. After successful fertilization in the ampulla of the fallopian tube, the zygote migrates towards the uterine cavity with the assistance of the ciliary motility of fallopian tube epithelium [5]. During this time, the zygote undergoes a sequence of cell divisions resulting in a multicellular structure termed the blastocyst. The blastocyst possesses an inner cell mass, which will form the embryo and an outer layer of cells called trophoblasts, which will develop into the placenta [6]. Prior to implantation the blastocyst moves freely within the uterine cavity. At the time of apposition and adhesion, the trophoblast cells begin to express selectins on its surface [2] which bind to the selectin ligands on the endometrial epithelial cells. In addition, the epithelial cells also express integrins, glycoproteins and other adhesion molecules which play a crucial role in ensuring proper attachment of the blastocyst to the luminal epithelium to initiate the implantation response [7].

### 2.2. Structure of the Endometrium

The endometrium is the inner layer of the uterus composed of epithelial cells, stromal cells, immune cells and endothelial cells which contribute to the vasculature of the uterus. The epithelial cells form a single layer of columnar epithelium (luminal epithelial cells) that faces the lumen of the uterus. Epithelial cells (glandular epithelium) which invaginate within the endometrial stroma, form distinct structures with a secretory function named endometrial glands. These glands are branched-tubular structures that develop through the endometrial stroma reaching the myometrium. The structure and function of these glands dynamically change throughout the menstrual cycle. During the peri-implantation period, glands achieve their peak functional role. The endometrial stroma is composed of connective tissue and extracellular matrix and it responds to hormonal influences, which alters its structural composition [8,9].

The endometrium can be differentiated into two regions: functionalis and basalis. The functionalis region responds to hormones, undergoes dynamic remodeling changes in cell morphology and function during the menstrual cycle. It sheds every month in the absence of a pregnancy and is the site of embryo implantation. The layer underneath, the basalis, does not shed but plays a role in the regeneration of the functionalis after menstruation [8]. During the 28 days of menstrual cycle, the endometrium undergoes remodeling under hormonal regulation to regenerate its cellular population to ensure its functionality.

In humans, the menstrual cycle (~28 days) starts with menstruation (days 0 to 4). The first part of the menstrual cycle (days 5 to 13 days) is known as follicular or proliferative phase. During this period, the endometrium thickens due to the rising levels of estrogen from ovarian follicles promoting the proliferation of the epithelium, endometrial glands and vasculature. In the middle of the cycle (day 14) ovulation occurs due to a surge of follicle-stimulating hormone (FSH) and luteinizing hormone (LH). The second part of the cycle is known as the luteal or secretory phase (days 15 to 28). This period is characterized by the control of the endometrium by progesterone in preparation for implantation. The imposition of progesterone over estrogen defines the “window of implantation” (days 20–24) during the secretory phase [1,10,11].

### 2.3. The Window of Uterine Receptivity

Successful embryo implantation requires a functional communication between a blastocyst and a receptive endometrium during a brief period of time known as the window of implantation [12,13].

During the window of implantation, the blastocyst can attach to the endometrial epithelial cells and invade the endometrial stroma and vasculature. This process can only occur when the endometrium is receptive [14]. During the secretory phase, a receptive endometrium is characterized by the appearance of microvilli on the apical surface of the luminal epithelial cells called pinopodes [15,16,17]. On average, pinopodes last for 1 or 2 days, usually during days 20 and 21 of the menstrual cycle. However, there is up to 5 days of variation between women in the timing of appearance [18]. Detection of pinopodes in the human endometrium is proposed as a clinical marker to assess uterine receptivity [19]. Studies have shown a correlation of the number of these structures with endometrial receptivity for blastocyst implantation in human [20,21]. Pinopode development during the mid-luteal phase is associated with an increase in expression of leukemia inhibitory factor (LIF) and its receptor, progesterone and integrin αVβ3 [17,22,23,24]. All these molecules are crucial in the communication between the blastocyst and endometrium.

## 3. Mediators of Implantation

The process of implantation in humans and primates involves a coordinated sequence of events that are critical for the establishment of pregnancy. There are many mediators under the control of ovarian hormones that are crucial during endometrial receptivity. Some of those mediators include cytokines, growth factors, cell adhesion molecules, amongst others [12].

### 3.1. Embryo-Derived: Chorionic Gonadotropin (CG)

Chorionic gonadotropin (CG) is one of the major embryonic signals in primates that modulates the uterine environment promoting uterine receptivity [25]. Its levels can be detected and measured in maternal serum 10 days after fertilization [26].

CG is a heterodimeric glycoprotein hormone. The thyroid stimulating hormone (TSH), follicle-stimulating hormone (FSH) and luteinizing hormone (LH) belong to the same family. Like CG, these hormones have a common α-subunit but differ on their β-subunit [27]. These subunits are held together by a non-covalent hydrophobic and ionic interactions [28]. Four different isoforms of human chorionic gonadotropin (hCG) have been described: hCG, hyperglycosylated-hCG (h-hCG), free beta-subunit-hCG and pituitary hCG. Each of them is produced by separate cells and differ in their functions [28]. Human CG, the most predominant form during pregnancy, is produced by villous syncytiotrophoblast cells. The villous syncytiotrophoblast produces and secretes hCG together with other placental hormones, such as placental lactogen and steroids, as the pregnancy advances [28,29]. 

One of the major functions of hCG is to promote the production of progesterone by the corpus luteum (CL) to maintain pregnancy while increasing its life span and rescuing it from regression [30]. It also plays an important role in angiogenesis and vasodilatation in the endometrium, promotes uterine growth simultaneous to fetal growth, suppresses myometrial contractions during pregnancy and promotes the maternal tolerance of the embryo by interacting with immune cells and modulating T cells during the implantation period [28,31]. Studies using the baboon model have shown the direct role of CG on endometrial receptivity by modulating endometrial stromal and epithelial cells [32,33].

In stromal cells, α-smooth muscle actin (α-SMA), which is expressed as part of their differentiation into a decidual phenotype, is directly regulated by CG. This regulation prevents stromal cell apoptosis and enables the process of decidualization [33]. Disruption of α-SMA not only leads to apoptosis, it also decreases Notch1, a Notch signaling receptor, which is induced by CG and promotes decidualization [30,34]. Studies in baboons have shown that the administration of hCG can induce endometrial stromal expression of α-SMA and Notch1 promoting cell survival and cell differentiation during decidualization [35,36]. Several studies have shown the relevance of the dysregulation of hCG in reproductive disorders related with infertility [37,38,39].

### 3.2. Cytokines

Cytokines are a group of proteins that are involved in the maternal-embryo interaction during the implantation process and regulate the immune adaptation and tissue remodeling. They play an important role in the adhesion of the blastocyst to the luminal epithelium, facilitating the physical contact between embryo and uterus and promoting placental development [40]. Implantation can be characterized as an inflammatory response and cytokines are responsible for this response.

#### 3.2.1. Leukemia Inhibitory Factor (LIF)

Leukemia inhibitory factor (LIF) has been demonstrated to be an important factor in relation to endometrial receptivity. Different studies have reported the maximal expression of LIF at the mid-secretory phase and plays a role during embryo attachment [41,42]. LIF is a pleiotropic cytokine that belongs to the IL-6 family having a four α-helix structure [43].

LIF regulates trophoblast cell adhesion and might be important for embryo invasion and placental development [25]. The uterine expression of this cytokine in the luminal epithelium, as previously mentioned, plays a crucial role during the attachment of the blastocyst and promoting the appearance of the pinopodes [23,44]. Women with recurrent implantation failure have shown a decrease in LIF production [45]. Studies have shown that mutations in the LIF gene may result in low levels of this cytokine reducing its activity in the endometrium and causing a high risk of implantation failure [44,46].

#### 3.2.2. Interleukin-6 (IL-6)

The Interleukin-6 (IL-6) family includes LIF and IL-6 cytokines, which are known for playing a role during embryonal development. IL-6 is a pleiotropic cytokine that is involved in the acute inflammatory response [47] but besides its immune role, IL-6 is also involved in processes related with fertility [48]. This cytokine is produced by a diverse type of cells including macrophages, fibroblasts, epithelial cells and placental trophoblasts [49,50].

IL-6 is a proinflammatory cytokine produced, mostly, by endometrial epithelium and stromal cells during the time of implantation [51]. It is produced in the luminal epithelium and the expression levels are the highest during the luteal phase, in the window of implantation, and menstruation [52]. Its expression in the endometrium changes in response to hormones. It increases during the mid to late secretory phase and decreases progressively during the late secretory phase [53,54]. IL-6 is expressed in gestational tissues and in the female reproductive tract. This cytokine not only plays a role during embryo implantation and placental development, it is also required to continue the pregnancy [47]. The expression of IL-6 during the window of implantation in the endometrium and the blastocyst emphasizes the relevance of IL-6 during the pre-implantation period [2].

#### 3.2.3. Interleukin-1 (IL-1)

The family of IL-1, relevant for their role in the inflammatory and immunological responses, is composed of three polypeptides: IL-1α, IL-1β and one receptor antagonist, IL-Ira. Although three of them are encoded and located in separate areas of chromosome 2, the three proteins recognize the same receptor, IL-1 receptor (IL-1R). IL-1α and IL-β produce similar biological effects [55,56].

IL-1 is known as one of the crucial paracrine factors that can modulate the cross-talk between the embryo and the maternal endometrium [56]. In the human endometrium, IL-1α and IL-β are omnipresent in the epithelium and stromal cells [57]. Studies using the baboon model have shown the relevance of IL-1β in affecting endometrial responses [58,59]. IL-1β is secreted by cytotrophoblast cells, having the highest expression during the first trimester of pregnancy [60].

Our laboratory has shown that in stromal cells, IL-1β can modulate changes in the cytoskeleton and induce the expression of cyclooxygenase-2 (COX-2) and metalloproteinase-3 (MMP-3). COX-2 synthesis is followed by an increase in prostaglandin E2 (PGE2) and intracellular cyclic adenosine monophosphate (cAMP), which in presence of steroid hormones, induces insulin-like growth factor binding protein-1 (IGFBP-1) expression in human and baboon stromal fibroblasts [61]. However, if cAMP is present simultaneously with IL-1, the differentiation and IGFBP-1 induction is inhibited. This suggests the negative cross talk between the two pathways and the relevance of maintaining an appropriate homeostasis for the process of decidualization and trophoblast invasion [61].

On the other hand, during decidualization, the extracellular matrix (ECM) is transformed and expresses additional proteins and new basal laminar components [62]. Active remodeling occurs during the implantation process and metalloproteinases, particularly MMP-3 seems to be crucial for this event. MMP-3 is secreted by cells in an inactive form, pro-MMP-3. After activation, it is capable of initiating ECM degradation that can disrupt the bidirectional signaling between integrins and the cytoskeleton, promoting a down-regulation of α-SMA during decidualization [58]. The studies in the baboon model suggest that IL-1β can contribute to the differentiation of the stromal cells by reorganizing the cytoskeleton and the indirect increase of cAMP. These changes are critical for IGFBP-1 expression and decidualization [58].

### 3.3. Celular Adhesion Molecules (CAMs)

The cellular adhesion molecules (CAMs) family includes members such as integrins, cadherins and selectins.

#### 3.3.1. Integrins

Integrins are a family of transmembrane heterodimeric glycoproteins that facilitate cell-extracellular matrix adhesion. They are formed by the association of a non-covalently linked α and β subunits [2]. They experience dynamic spatial and temporal changes in the endometrium during the menstrual cycle [62]. They are also expressed in the human trophoblast during the time of implantation [63].

Their major roles are focused on differentiation, apoptosis, motility and attachment [64]. During implantation, integrins play a role during the attachment of the cells to the ECM and initiating a signaling transduction from the embryo to the ECM to initiate the translation of genes involved in the implantation process [65]. There are different isoforms in mammals, but the α1β1, α4β1 and αVβ3 are the most relevant during implantation. In humans, these three isoforms are expressed in the endometrium during the window of implantation, when the endometrium is structurally and physiologically responsive to blastocyst implantation [66,67].

Integrins are considered to be excellent markers of uterine receptivity. During decidualization, there are specific changes in integrin expression [22,68]. Similar changes in integrin expression were also observed in our baboon model [62] during the menstrual cycle and pregnancy [22].

#### 3.3.2. Cadherins

Cadherins are a group of calcium-dependent glycoproteins that are responsible for cell-to cell-adhesion. E-cadherin, a member of the cadherin family, is expressed by different tissues and plays a crucial role during mammal development and pre-implantation stages [69,70].

This transmembrane protein is highly expressed in the cytotrophoblast restraining its invasiveness. At the moment that the syncytiocytotrophoblast begins to differentiate into a trophoblast, E-cadherin starts its down-regulation enabling the epithelial cell dissociation and promoting the invasion of the blastocyst [2,71]. There is evidence that E-cadherin can contribute to the invasiveness and motility of the trophoblast during implantation [69].

#### 3.3.3. Selectins

Selectins are glycoproteins with a single chain transmembrane domain and a small cytoplasmatic tail. The three known members of this family are P-selectin, L-selectin and E-selectin [72]. L-selectin is the most relevant during the implantation process [73].

Studies have demonstrated the relation between L-selectin ligands and the implantation process. MECA-79, a specific endothelial L-selectin ligand antibody, was observed to change its expression in the epithelium of the human endometrium from a non-receptive phase to a receptive phase [74]. After attachment, the trophoblast cells begin invading into the decidua. At this moment, the expression of L-selectin ligands is shifted to decidual cells indicating a role in the adhesion and progressing penetration of the cytotrophoblast into the decidua [74]. These studies show that L-selectins and L-selectin ligands play an important role during the window of implantation.

Defects in L-selectin adhesion could explain causes of infertility, early pregnancy loss or insufficient cytotrophoblast invasion which could be related with pregnancy difficulties [74,75].

### 3.4. Mucin-1

Mucin-1(MUC-1) is a highly glycosylated transmembrane glycoprotein expressed at the apical surface of the endometrial epithelium [76]. In women, MUC-1 expression is up-regulated in the secretory phase and remains high during the receptive period, when embryo implantation occurs [77]. Its high degree of glycosylation prevents degradation and provides protection from proteolysis [76]. However, this glycoprotein could act as a barrier to embryo implantation in other species of animals [78]. Studies in the baboon model have shown that MUC-1 displays a strong surface expression on days 5–8 after ovulation which could promote uterine resistance to microbial challenge introduced during copulation. Its expression decreases during the receptive phase [79].

The expression of MUC-1 is progesterone dependent. Progesterone combined with estradiol induces an up-regulation of MUC-1 in the receptive endometrium. MUC-1 is removed from the apical endometrium just at the time of implantation in the baboon. Removal of this protein from the epithelial surface at the implantation sites is performed by signals produced by the blastocyst [78]. In the baboon, uterine epithelial MUC-1 is also up-regulated by progesterone, but there is a differential expression of MUC-1 between luminal and glandular epithelia [79].

## 4. Dynamics of Implantation

During the window of implantation, the endometrium expresses several genes that enable the process of implantation to occur. The uterus undergoes extensive tissue remodeling that share similarities with a micro metastasis process [3].

**Figure 1:**
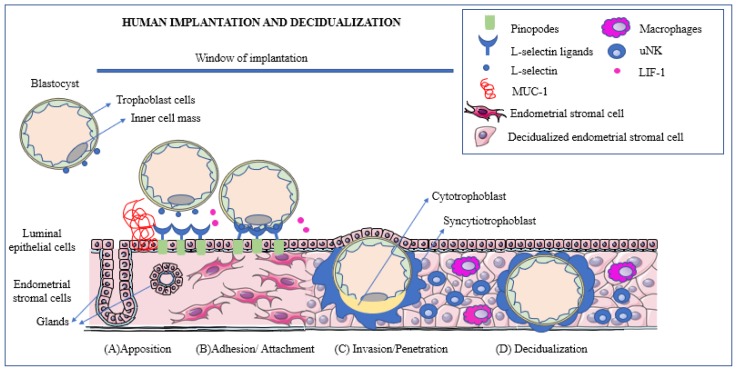
Human implantation is a process that could be divided into apposition, adhesion/attachment and invasion/penetration. During apposition (**A**), the blastocyst expresses L-selectins. The presence of Mucin-1 (MUC-1) repels the blastocyst and prevents it from attaching outside of the window of uterine receptivity. The L-selectins interact with the L-selectin ligands, which are expressed mainly on the pinopodes during the implantation window. At the beginning of the adhesion phase (**B**), the blastocyst promotes the cleavage of MUC-1 at the implantation site to ensure successful attachment. Cytokines such as Leukemia inhibitory factor (LIF), play an important role during human implantation by supporting the embryo-endometrial interactions. During the invasion or penetration phase (**C**), the trophoblast cells from the blastocyst penetrate the endometrial epithelium into the stroma. The extra-villous trophoblast cells start proliferating and differentiate into inner cytotrophoblast and outer syncytiotrophoblast. Once implantation is initiated and the embryo breaches the luminal epithelium, the stromal cells surrounding the embryo transform into decidualized cells (**D**). Immune cells such as macrophages and uterine natural killer (uNK) cells play an important role during decidualization to promote an environment that is conducive to successful implantation. Some art elements used in this figure were obtained from Servier Medical art (http://smart.servier.com). Servier Medical Art by Servier is licensed under a Creative Commons Attribution 3.0 Unported License.

Upon entry into the uterine fundus, the blastocyst establishes its adherence to the apical surface of the epithelium and penetrates the luminal epithelium invading the stroma [80,81,82]. As mentioned previously, implantation could be divided into different phases: apposition, adhesion/attachment, invasion/penetration and immune regulation [2,3] (Figure 1).

### 4.1. Embryo Implantation

#### 4.1.1. Apposition

Once the endometrium is receptive (window of implantation) and the blastocyst enters the uterus, a loose interaction occurs between the blastocyst and the luminal epithelium of the endometrium. Communication between blastocyst and uterus is established and apposition is the first connection between blastocyst and endometrium [82]. During this stage, receptor-ligand interactions are critical. The blastocyst enters into the uterus, rolling freely over the endometrium expressing adhesion molecules such as L-selectin [74]. Selectins play an important role in this step ensuring the rolling and tethering of the blastocyst. The human embryo needs to align to the receptive endometrium with a specific inner cell mass orientation to ensure a proper apposition. These selectins mediate the apposition of the blastocyst into the uterine epithelium interacting with L-selectin ligands [9,13,83], which are mainly detected on pinopodes where blastocyst adhesion is initiated [84]. The implanting embryo also encounters a glycocalyx associated with the luminal epithelium that contains different adhesion molecules. One of them is MUC-1, identified as an anti-adhesion molecule [78]. The purpose of MUC-1 at this stage is to prevent the blastocyst from binding to an area with poor chances of implantation. Its expression increases just before implantation to prevent the embryo attaching in the wrong location [2].

#### 4.1.2. Adhesion/Attachment

Removal of the pre-existing layer of mucins is necessary for blastocyst adhesion. During the apposition stage, the presence of the blastocyst promotes the increase of levels of MUC-1 in the luminal epithelium, but, at the beginning of the adhesion phase, the blastocyst induces the cleavage of MUC-1 at the implantation site to promote successful attachment [78]. Several chemokines and cytokines are essential during the process of adhesion. One of their functions is to attract the blastocyst to the location of implantation. The most relevant cytokine for implantation and the most studied is LIF [85]. This cytokine plays an important role during human implantation [86]. In addition to this, studies have shown the co-expression of LIF and pinopodes. In the human endometrium, expression of LIF reaches maximal levels during the mid-secretory phase, during which the endometrium is under the influence of progesterone [23]. Clinical studies have shown that LIF deficiency may be associated with infertility in women, which shows its relevance during implantation [87]. Adhesion molecules such as integrins, are also necessary to attach the blastocyst to the pinopodes to ensure a firm implantation [80,88]. Among these integrins, the heterodimer αVβ3 is crucial for endometrial recognition during the adhesion. It is expressed in the human trophoblast cells and uterine luminal epithelium during implantation and participates in endometrial recognition [89]. Studies have shown that abnormal expression of this integrin could be associated with cases of recurrent pregnancy loss and infertility [90].

#### 4.1.3. Invasion/Penetration

During invasion or penetration, trophoblast cells from the blastocyst penetrate the endometrial epithelium invading the underlying endometrial stroma with the purpose of reaching maternal blood vessels [91]. Trophoblast cells start developing thin folds, named invadopodia, that grow between adjacent endometrial epithelial cells. They are intended for the degradation of the basement membrane, allowing the trophoblast cells to spread into the endometrial stroma [92,93]. Trophoblast cells proliferate and differentiate into inner cytotrophoblast and outer syncyotiotrophoblast. The syncyotiotrophoblast invades the luminal epithelium, which is called syncytialization [94]. In humans, the embedding of the blastocyst within the stroma is completed 8 days after ovulation occurs. The entry site is then covered with fibrin and the syncyotiotrophoblasts fluid-filled spaces separate by trabeculae, appearing to transform the syncyotiotrophoblast into a spongy material [93]. The trabeculae are arranged radially, and cytotrophoblastic cells proliferate within the trabeculae, forming a primary chorionic villus. Over time, the primary villi grow and branch into secondary and tertiary villi. This process is known as placentation [93].

### 4.2. Decidualization

Once implantation occurs and the embryo breaches the luminal epithelium, the stromal cells surrounding the implanting embryo transform into a decidua by a process called decidualization [9,95].

#### 4.2.1. Definition

Decidualization is the transformation that the uterine stromal cells undergo to accommodate the embryo while establishing a successful pregnancy. This process is characterized by the differentiation of the endometrial stromal cells (elongated fibroblast-like cells), into a decidual cells (rounded epithelial-like cells), during the menstrual cycle and pregnancy [96,97]. In humans, this process is initiated in the mid-secretory phase of each menstrual cycle as a result of elevated levels of progesterone. If pregnancy occurs, the elevated levels of progesterone will maintain the decidua to assure an ongoing pregnancy [96,98].

#### 4.2.2. Cellular Composition of Decidual Stroma

The main cell type of the decidua is the uterine stromal cells. Stromal differentiation and angiogenesis during decidualization are essential for the establishment and maintenance of pregnancy [82,99]. Apart from the decidual stromal cells, the endometrium hosts a dynamic population of cells, including hematopoietic cells that can play a role in implantation, but also in absence of pregnancy and menstruation [100]. Macrophages, lymphocytes and decidual leukocytes also play a role during decidualization [100]. Decidual leukocytes not only play a role in providing maternal immune tolerance, they also contribute to decidual remodeling during pregnancy [101]. Among these leukocytes, the uterine natural killers (uNK) are the most involved in the maternal immune tolerance (70% of the decidual immune cells). They are present in the human endometrium across the cycle and become activated and dramatically increase during decidualization [102,103]. They are abundant around spiral arteries, endometrial glands and adjacent to the growing conceptus to support the maternal blood supply [104]. Monocytes are the second largest component of the leukocyte population within the decidua [100]. Studies have observed an increased infiltration of monocytes between 7 and 20 weeks of gestation during pregnancy [105].

#### 4.2.3. Tissue Remodeling and Transformation

Decidualization of the endometrium is an event that occurs in species in which placentation involves breaching of the luminal epithelium and invasion of maternal tissues by the trophoblast [106]. Decidualization is the reprograming of the endometrial stromal cells into secretory epithelial-like cells [95]. The endometrium transforms into a vascularized receptive tissue characterized by the proliferation of differentiated stromal decidual cells, increased vascular permeability, invasion of leukocytes, vascular remodeling and angiogenesis. An important feature of decidua is its function in controlling trophoblast invasion. Invasion is permitted to access the maternal blood supply, but not to the extent of endangering the mother [107]. During implantation, the decidua differentiates into different regions: decidual basalis underneath the implantation site, decidua parietalis adjacent to basalis and the more distant, decidual secretory endometrium which remains similar to the pre-decidualized endometrium. These differentiations occur due to the presence of hormones such as hCG, estradiol and progesterone immune cells and the trophoblast during the early stages of pregnancy [108]. Immune cells also play a role during the formation of the decidua. Leukocytes infiltrate into the endometrium in response to hormonal signaling occurring during the invasion [109]. Apart from leukocytes, other components such as macrophages, growth factors and cytokines are expressed by uNK cells that facilitate and control the invasion of the trophoblast cells, while promoting vascular transformation [110]. The cytotrophoblast cells develop anchoring systems that promote the interaction with the decidual stromal cells, glands and the maternal immune system. The trophoblast moves towards the maternal blood vessels under the control of hormonal and cytokine signaling [111]. Angiogenesis and vasculogenesis change the decidua with the purpose of initiating the development of the placenta and to coordinate an independent (embryo-mother), vascular system [112].

#### 4.2.4. Role of Notch during Decidualization

The Notch pathway is one of the most highly conserved signaling cascades in multicellular organisms and plays an important role in morphogenesis and homeostasis in adult and embryonic tissues [113,114]. Notch signaling consists of four transmembrane receptors (Notch 1 to Notch 4) that interact with five transmembrane ligands (Delta-like or Jagged-like) on adjacent cells [36,115]. In the canonical Notch signaling, the Notch intracellular domain (NICD), which is the active form of Notch, translocates to the nucleus binding and activating protein-Jk (RBPJ). Following that, Notch target genes, such as Hairy enhancer of split (HES) and Hes-related (HEY) transcription factor families activate [116,117].

In the human endometrium, the Notch1-3 receptors are located in epithelial and stromal cells. Ligands Jagged1 (Jag1) and Delta-like 4 (DLL4) are primarily present in epithelial cells [118]. Studies in the baboon model have shown the expression of Notch1 during the secretory phase in endometrial stromal cells [36]. It is known that Notch signaling plays a crucial role during the decidualization process. The expression of Notch1 and its target, α-SMA, are enhanced by CG from the implanting blastocyst and progesterone. In vivo infusion of CG in the baboon model has shown the upregulation of Notch1 and α-SMA [36].

Progesterone also plays an important role in the Notch1 regulation. Progesterone, along with CG activate Notch1, which promotes α-SMA expression and inhibits apoptosis in stromal cells by initiating their differentiation into decidual cells. These findings suggest that Notch signaling promotes proliferation, differentiation and apoptosis in the primate endometrium [30]. In vitro studies using human uterine fibroblasts (HUF) have demonstrated that the silencing of Notch1 before the induction of decidualization inhibits this process decreasing the levels of IGFBP-1 [117]. It also has been observed that levels of Notch1 are significantly decreased in women with endometriosis and baboons with spontaneous diseases compromising the fertility of the females [117]. Notch1 plays a key role during the transformation of stromal fibroblast into decidual cells. Any failure in Notch1 signaling could result in subsequent impaired decidualization compromising pregnancy.

## 5. Role of MicroRNAs during Embryo-Maternal Dialogue

### 5.1. Biogenesis of MicroRNAs

MicroRNAs (miRNAs) are small noncoding regulatory RNAs that are not translated into proteins [119]. MiRNAs contain ~20 nucleotides that can regulate gene expression and play a fundamental regulatory role in several pathological processes [120,121,122]. In 1993, Rosalind Lee, Rhonda Feinbaum and Victor Ambros presented the first evidence of what it is now known as microRNAs. Ambros’s group described a 22 nucleotide RNA encoded by lin-4, a gene in Caenorhabditis elegans involved in larval development that does not code for a protein, but instead can bind to the lin-14 transcript and regulate its expression [120,122,123]. In the canonical miRNA biogenesis pathway, miRNAs genes are transcribed by RNA polymerase II or RNA polymerase III to produce a primary microRNA transcript (pri-miRNA). This transcript, still in the nucleus, is processed into a smaller precursor miRNA (pre-miRNA), by the microprocessor complex Drosha-DGR8. The resulting precursor is translocated from the nucleus to the cytoplasm by Exportin-5-RAN-GTP. Once there, Dicer-TRBP complex cleaves the pre-miRNA into a mature single-stranded miRNA. The mature miRNA binds to its messenger RNA (mRNA) target at their complementary sequence to reduce the expression of their target protein by inhibiting mRNA translation to proteins or simply by decreasing the mRNA levels [119,124,125]. The study of miRNAs is crucial for the understanding of the pathophysiology of different diseases.

### 5.2. MicroRNAs during Embryo Implantation

The process of implantation involves a complex crosstalk between maternal cells and the developing embryo. Different cell types have the potential to secrete miRNAs contained within extracellular vesicles into the uterine fluid to facilitate the maternal-embryo communication. Numerous studies have observed the presence of miRNAs during the reproductive process [126]. They play a role in fertilization, implantation and placentation. Studies have shown that altered expression of miRNAs involved in endometrial receptivity and embryo implantation could cause implantation failure [127]. In addition, different studies have reported altered expression of miRNAs associated with gynecological diseases such as polycystic ovarian syndrome [126] and endometriosis in humans [127,128] and in the baboon model [129,130].

Ovarian hormones such as hCG, estrogen and progesterone play a crucial role in establishing and maintaining pregnancy. Progesterone and estrogen receptors can interact with miRNAs at the transcriptional and translational level contributing to the implantation process [131]. Studies have demonstrated the presence of miRNAs in placental tissues and immune cells suggesting that they are likely to be universally involved in pregnancy, placentation, immune tolerance and angiogenesis at the maternal-fetal interface [132].

There are miRNAs that participate in uterine events during the implantation process. For example, miR-199 and miR-346 can target LIF affecting uterine receptivity [133]. High levels of miR-145 have been associated with suppression of embryo attachment by regulating type-1 insulin-like growth factor receptor (IGF1R). Patients with repeated implantation failure have been identified with elevated miR-145 [134]. Stromal cells decidualize, in response to penetration of the trophoblast, triggering massive proliferation and differentiation in humans and baboons.

Impaired decidualization and embryo-maternal interactions have been associated with an increased expression of miR-29c observed in the baboon model of endometriosis [130].

In addition to this, it has been shown that miR-181a stimulates the expression of genes related with decidualization such as forkhead box O1 (FOXO1), prolactin (PRL), IGFBP-1, inducing a morphological transformation of the cells. The inhibition of this miRNA causes an impaired induction of the decidual reaction [135].

In vitro studies have shown that secretion of miRNAs can come from trophoblast cells and primary human trophoblast [136] from the developing embryo but also, they could have maternal origin. These secreted miRNAs could be involved in the modification of transcriptomes in order to facilitate implantation.

## 6. Disruption of Embryo Implantation

Successful implantation requires a receptive endometrium, a functional embryo and a synchronized dialogue between them [12,13]. Uterine receptivity plays a crucial role in this process during the window of implantation. Under certain anatomic or inflammatory conditions, such as endometriosis, the window of implantation can be affected preventing normal implantation which could lead to infertility or pregnancy loss [14]. Implantation can also be disrupted in the setting of a structurally abnormal uterine cavity. Some of the intrauterine problems that could decrease embryo implantation are:

### 6.1. Structural Defects: Polyps and Fibroids

Polyps are identified in 8–12% of women in reproductive age [137,138]. Endometrial polyps are defined as endothelial tumors comprising of endometrial glands, stroma, blood vessels and fibrous tissue. Their size can vary from millimeters to centimeters, and they are commonly found during hysteroscopy [138]. There is one randomized study in this field that demonstrated the relationship between polypectomy (therapeutic procedure to remove polyps) and infertility. The results showed an increase of pregnancies in women who underwent polypectomy [139]. More research in this field would help to further understand the relationship between polyps and their impact during implantation.

Fibroids are generally found in almost 70% of Caucasian women, and 80% of African-American women [140]. Fibroids have been noted more frequently in women with infertility, however most of the women with fibroids are fertile [141]. Fibroids may be involved more in the anatomical distortion of the uterine environment. Studies have shown evidence of impaired endometrial receptivity due to the presence of fibroids [142]. In IVF treatments it has been shown that submucosal and intramural fibroids that invade the endometrial cavity are associated with a decreased rates of implantation [143].

### 6.2. Endometriosis

Endometriosis affects 10% of women in reproductive age [144]. It is defined as an estrogen-dependent disease that is characterized by the presence of endometrial tissue outside the uterine cavity, primarily in the peritoneal cavity and ovaries. The main clinical features of endometriosis are pelvic pain and infertility [145,146]. Infertility could be caused by the physical blockage of the fallopian tubes, but also by the decreased expression of implantation markers during the window of receptivity [147]. One of the treatments for endometriosis to remediate infertility is the surgical removal of endometriotic tissue or assisted reproductive technology [148]. Studies involving the comparison of gene expression profiling of endometrium from women with and without endometriosis have revealed candidate genes related to the failure of implantation and altered steroid hormone pathways [149].

Around 25 to 50% of infertile women have endometriosis, and 30 to 50% of women with endometriosis are infertile [150]. Although research in endometriosis is advancing, it is not clear the mechanisms that could explain the association between endometriosis and infertility. Possible mechanisms by which endometriosis could cause infertility are uterine anatomical distortion, poor oocyte quality, fallopian tube and embryo transportation, diminished ovarian reserve and compromised endometrial receptivity [151]. Furthermore, there are endometriosis patients that present progesterone resistance. Progesterone resistance plays an important role in impaired decidualization. It is very well established in endometriotic lesions and eutopic endometrium of women with this disease [152]. Transcriptional regulators such as forkhead box O1 (FOXO1), AT-rich interaction domain 1A (ARID1A) and histone deacetylase 3 (HDAC3) are associated with progesterone signaling [153,154,155]. These factors are down regulated in women with infertility associated with endometriosis. Studies have shown that ARID1A has a crucial role in implantation and decidualization, since ARID1A expression is lost in endometriosis [153].

As previously described, miRNAs can play a role during the implantation process, but they also are critical in the progression of endometriosis affecting infertility. Studies have shown the relevance of miR-29c in progesterone resistance. This miRNA is upregulated in endometriosis decreasing the expression of one of its targets, FK506-binding protein 4 (FKBP4) gene. FKBP4 is a co-chaperone that optimizes the function of the progesterone receptor [156]. The decrease or absence of this gene results in a weak progesterone response promoting defective decidualization and implantation in humans and baboons [130]. The alteration of miR-29c resulting in the decrease of FKBP4 during the window of implantation could lead to the progesterone resistance in women with endometriosis, promoting infertility [156]. These in vivo experiments have been further characterized in vitro using HUF cells and showing a compromised decidualization response compared to positive controls [130].

In a different study, the overexpression of miR-29c was reported in women with ovarian endometriosis, showing in this case an increase of specific extracellular genes promoting the dysregulation of the uterine function including the decidualization response [157].

Besides miRNAs, Notch1 signaling is also affected during endometriosis. Studies have demonstrated that Notch1 signaling is significantly decreased in eutopic endometrium of women and baboons with the disease compared with the controls (disease free). This decrease in Notch signaling has also been observed in in vitro experiments using endometrial stromal cells from women with the disease and showing a substantial defective decidualized response. These results could be caused in part by the decreased expression of one of the Notch targets, FOXO1 which inhibits the decidualization response [117].

## 7. Consequences of Defective Implantation: Clinical Relevance

The fundamental role of the endometrium is to support the implanting embryo and provide nourishment during pregnancy. Implantation and decidualization are essential for an ongoing pregnancy. Any major aberration during the process could either terminate or carry defects during pregnancy.

### 7.1. Recurrent Miscarriage and Implantation Failure

Miscarriage or spontaneous pregnancy loss affects 15–18% of couples [158] being approximately 1–3% of women who have recurrent miscarriage [159]. A pregnancy loss (miscarriage) is defined as the spontaneous demise of a pregnancy before the fetus reaches viability. This includes all pregnancy losses from the time of conception until 24 weeks of gestation. By definition, “recurrent” pregnancy loss or miscarriage is defined as the loss of two or more pregnancies [160].

Interference during the angiogenesis and the dysregulation of angiogenic factors may play a crucial role in the pathogenesis of miscarriages. An increase in the blood vessel density within decidua parietalis could be associated with spontaneous miscarriage [158]. Recurrent implantation failure is determined when high-quality embryos transfers fail to implant after in vitro fertilization (IVF) [161]. Mechanisms by which this event may happen include a decrease in endometrial receptivity which could be associated with uterine abnormalities or diseases such as endometriosis [159]. Several studies suggest that an imbalance of the immune function could play a role in women with recurrent miscarriage and implantation failure [162].

The pregnancy-related changes in the endometrium related to a successful implantation include important changes in the levels of immune cells including macrophages, uNK and a distinctive cytokine profile [159,162]. Studies have shown that an increased accumulation of uNK cells in the decidual secretory endometrium could result in an increased risk of miscarriage [163]. This increase in cell density could participate in the increased pre-implantation angiogenesis leading to a premature contact with the maternal circulation promoting the miscarriage [164].

In addition to this, cytokine production by natural killers (NK) seems to be dysregulated in women with recurrent miscarriage and implantation failure. Pro-inflammatory cytokine levels are higher in healthy pregnant women when compared with non-pregnant women, but the levels appear to be considerably higher in women with recurrent miscarriage [165].

The overall picture suggests that although an inflammatory reaction is critical for pregnancy, the cytokine imbalance may lead to a recurrent miscarriage.

### 7.2. Pre-eclampsia

Hypertensive disorders during pregnancy affect 10% of pregnant women. Pre-eclampsia is a disorder that affects 3–5% of all the pregnancies with hypertensive disorders [166]. This disease is not fully understood, but it involves dysfunctional placentation, systemic inflammation and oxidative stress [167]. Aberrant decidualization can result in an adverse pregnancy phenotype, including defects in placentation and parturition. Poor trophoblast invasion into decidua is one of the potential causes for pre-eclampsia [168]. In addition to this aberrant trophoblast migration, an altered remodeling of decidual arterioles could be associated with pre-eclampsia [169].

Bioinformatic studies have also shown differentially expressed genes in chorionic villous samples from women who developed severe pre-eclampsia as compared with normal pregnancies. A large number of the genes (40%) were related with different stages of endometrial decidualization, suggesting that insufficient or defective decidualization before and during early pregnancy could contribute to the development of pre-eclampsia [170].

Several studies have also shown that women with pre-eclampsia could experience an imbalance of cytokine production by NK [171].

## 8. Conclusions

Normal pregnancies include events such as uterine receptivity, attachment, decidualization and placentation. The order of these events is carefully orchestrated to promote pregnancy success. If the sequence of any of these events during implantation are unsuccessful or inadequately regulated, it may result in an abnormal pregnancy or abnormal depth of invasion, resulting in clinical implications such as placenta previa or ectopic placentation. Furthermore, it may result in pre-eclampsia or pregnancy loss. Premature decidual senescence can lead to preterm birth and fetal death, whereas shallow trophoblast invasion into maternal decidua and/or blood vessels could lead to pre-eclampsia. The function of the decidua is not only to establish and maintain pregnancy, but also to ensure immune tolerance towards the implanting blastocyst and protect it from the mother’s immune system [172].

## 9. Future Perspectives

Understanding the processes and mechanisms required for implantation could help to prevent adverse pregnancy outcomes during high risk pregnancies. Since the manipulation of human embryo or studies with pregnant women to understand these mechanisms is not possible, novel in vitro approaches are necessary. The development of functional in vitro systems to study embryo–uterine interactions will lead to a better understanding of the interactions between molecules involved in this process. Despite the importance of the endometrium as the site of implantation and nutritional support for the embryo, there are no long-term culture systems that recapitulate endometrial function in vitro. Organoid cultures which generate three-dimensional structures of normal and decidualized human endometrium are becoming more valuable as a long-term in vitro option [173]. Having a model to study uterine functions will increase therapeutic options to treat endometrial dysfunction and to better understand the physiology of early pregnancy.
